# Structures of the Ets Protein DNA-binding Domains of Transcription Factors Etv1, Etv4, Etv5, and Fev: DETERMINANTS OF DNA BINDING AND REDOX REGULATION BY DISULFIDE BOND FORMATION*

**DOI:** 10.1074/jbc.M115.646737

**Published:** 2015-04-12

**Authors:** Christopher D. O. Cooper, Joseph A. Newman, Hazel Aitkenhead, Charles K. Allerston, Opher Gileadi

**Affiliations:** From the Structural Genomics Consortium, University of Oxford, Old Road Campus Research Building, Roosevelt Drive, Oxford OX3 7DQ, United Kingdom

**Keywords:** DNA methylation, ETS transcription factor family, protein structure, protein-DNA interaction, redox regulation, dimerization

## Abstract

Ets transcription factors, which share the conserved Ets DNA-binding domain, number nearly 30 members in humans and are particularly involved in developmental processes. Their deregulation following changes in expression, transcriptional activity, or by chromosomal translocation plays a critical role in carcinogenesis. Ets DNA binding, selectivity, and regulation have been extensively studied; however, questions still arise regarding binding specificity outside the core GGA recognition sequence and the mode of action of Ets post-translational modifications. Here, we report the crystal structures of Etv1, Etv4, Etv5, and Fev, alone and in complex with DNA. We identify previously unrecognized features of the protein-DNA interface. Interactions with the DNA backbone account for most of the binding affinity. We describe a highly coordinated network of water molecules acting in base selection upstream of the GGAA core and the structural features that may account for discrimination against methylated cytidine residues. Unexpectedly, all proteins crystallized as disulfide-linked dimers, exhibiting a novel interface (distant to the DNA recognition helix). Homodimers of Etv1, Etv4, and Etv5 could be reduced to monomers, leading to a 40–200-fold increase in DNA binding affinity. Hence, we present the first indication of a redox-dependent regulatory mechanism that may control the activity of this subset of oncogenic Ets transcription factors.

## Teaser

**Background:** Transcription factors of the Ets family regulate development and cancer.

**Results:** We show the details of DNA sequence recognition and identify structural changes that drastically affect DNA binding.

**Conclusion:** The activity of Ets transcription factors is regulated by their oxidation state.

**Significance:** Ets transcription factors may control the response of cells to oxygen levels.

## Introduction

The Ets transcription factor family consists of 28 genes in humans (1, 2) containing the evolutionarily conserved 85 amino acid Ets DNA-binding domain (3), originally identified as a viral oncogene (E26 transformation-specific) (4). Ets proteins exhibit ubiquitous or tissue-specific expression (5) and are particularly involved in differentiation processes and the response to signaling pathways (6, 7). Ets proteins may be classified based on the presence of additional structured domains (5). The small PEA3 subfamily is characterized by an N-terminal transactivation domain and consists of three members, Etv1 (Ets translocation variant 1, ER81), Etv4 (PEA3), and Etv5 (ERM) (8).

PEA3 transcription factors have roles in morphogenesis (9) and neuronal differentiation (10, 11). PEA3 members are also oncoproteins (8), whose overexpression correlates with up-regulation of HER2/Neu and with progression in breast tumors (12). *ETV1* is amplified in >40% of melanomas (13), and overexpression following chromosomal translocation of either *ERG* or *Etv1* to the androgen-inducible *TMPRSS2* promoter is present in most prostate tumors (14). Etv1 directly interacts with the androgen receptor (15) and drives the androgen receptor transcriptional response associated with aggressive prostate cancer (16). Etv1 target genes include *hTERT* (17) and matrix metalloproteinases (18) that may mediate cancer development. Furthermore, Etv1 may be subverted by other oncoproteins such as mutated (activated) KIT, which cooperates with elevated Etv1 levels to promote tumorigenesis (19). Etv4 and Etv5 also play roles in morphogenesis, fertility, and oncogenesis (8). Ewing's sarcomas result from chromosomal translocations that generate dominant transforming fusion proteins of the transactivation domain of the EWS protein with the ETS domain of one of the five Ets proteins Etv1, Etv4, Erg, Fli1, and Fev (Fifth Ewing Variant, PET-1) (20, 21). Fev lacks additional domains and has a restricted tissue expression (21, 22). Fev regulates serotonergic neuronal differentiation, being critical for normal anxiety/aggression development (23), although overexpression is associated with serotonin production in small intestine neuroendocrine tumors, stimulating tumor growth (24). Ets domains are thus a central nexus in tumor development and disease progression. Targeting transcriptional regulation in cancer with drugs is expected to be challenging (25), but Ets inhibitors have been developed. YK-4-279 targets EWS-FLI1, inhibits growth in Ewing sarcoma (26), and also inhibits Erg/Etv1-driven prostate cancer invasion (27). Although this may be useful for generic treatment of Ets-driven cancers, the lack of specificity could cause off-target effects with other Ets proteins, highlighting the need for further high resolution structural and biochemical studies of Ets proteins.

The Ets domain is a variant helix-turn-helix (winged helix) structure (28–31), comprising three α-helices and a four-stranded antiparallel β-sheet. Ets domains bind to the EBS^5^ (Ets-binding site) in dsDNA with the α3 helix inserted into the major groove, and invariant arginine and tyrosine residues hydrogen bonded to bases of the invariant 5′-GGA(A/T)-3′ sequence in the EBS (2). Although binding the GGA(A/T) core is a common property of Ets transcription factors, a genome-wide analysis of all Ets family members has established that Ets domains recognize up to nine bases (32) (three upstream and two downstream of the GGA(A/T) core), and they are classified into four distinct classes based on the sequence preferences for these flanking regions. Etv1, Etv4, Etv5, and Fev all belong to the large and diverse class I (containing 14 of 26 of the mammalian Ets transcription factors), which recognize a consensus sequence ACCGGAAGT(G/A). The structural basis for this sequence discrimination is unclear, as there are no direct base contacts outside of the core. Indirect readout mechanisms have been suggested, based on sequence-dependent DNA conformational preferences (33). However, no mechanism for how sequence preferences would influence shape readout has been proposed nor how the different classes of Ets domains achieve their observed sequence specificities outside the GGA within this paradigm.

A number of other factors influence Ets DNA binding, including interaction with other transcription factors to recognize combined operators in a cooperative manner (34), cooperative binding of palindromic sequences by dimerization (35), and the presence of auto-inhibitory regions surrounding the Ets domain (36–38). DNA binding is also subject to post-translational regulation (39) with protein kinase A phosphorylation of Etv1 at Ser-334 (40) and the equivalent Ser-367 in Etv5 (41) repressing DNA binding. Several Ets proteins have been identified as being subject to redox control, with redox-sensitive cysteines present in GA-binding protein α (GABPα) affecting dimerization and DNA binding (42). However, as with the case for phosphorylation, the structural mechanism for this regulation is unclear.

Additional regulation of Ets DNA binding may occur at the DNA level, with several Ets motifs being over-represented in methylated genomic regions (43). Some Ets proteins are known to bind to their cognate sequence *in vitro* and *in vivo* only in a demethylated state, including GABPα (44, 45) and ETS1 (46). Direct methylation of the class I EBS sequence is possible in two positions (C^m^GGAA/TTCC^m^G); however, it is not known whether and to what extent this direct methylation affects Ets binding.

Here, we present the crystal structures of the Ets domains of the entire PEA3 subfamily (Etv1, Etv4, and Etv5) together with the structure of the Erg family member (Fev). All proteins share a highly conserved core Ets structure and make extensive interactions with DNA. Comparisons of apo- and DNA-bound forms of Etv1, Etv4, and Fev allow us to identify residue movements and disorder to order transitions that occur upon DNA binding. In the DNA complex structures of Etv5 and Fev (which are determined to 1.9 and 2.0 Å resolution, respectively), we observe a network of coordinated water molecules contributing to sequence recognition upstream to the GGA core. Structural and biochemical analysis of Etv1 clarifies mechanisms of post-translational regulation in Ets proteins, including PKA-mediated phosphorylation at Ser-334 and the abrogation of Ets binding to sites that include a methylated CpG. Furthermore, a new Ets dimerization interface linked by a redox-sensitive disulfide bond is identified, and this dimerization is shown to result in a significant inhibition of DNA binding, potentially linking Ets transcription factors with cellular redox regulatory mechanisms.

## Experimental Procedures

### 

#### 

##### Cloning and Site-directed Mutagenesis

Plasmid DNA templates for full-length Etv1, Etv4, Etv5, and Fev were obtained from the Mammalian Gene Collection (I.M.A.G.E. Consortium Clone IDs 30345383, 3854349, 3050350, and 4130242, respectively) (47). Regions corresponding to the core Ets domains were amplified by PCR using Pfx DNA polymerase (Invitrogen, Paisley, UK). PCR products were ligation independent cloning into the pNIC28-Bsa4 expression vector (GenBank^TM^ accession number EF198106, encompassing a tobacco etch virus (TEV)-cleavable (shown by *) N-terminal His_6_ tag MHHHHHHSSGVDLGTENLYFQ*SM), as described elsewhere (48). The initial Etv1 construct contained two primer-incorporated mutations (Y329S and P427S) and thus were designated Etv1^Y329S-P427S^. A subsequent construct containing the wild-type sequence is referred to as Etv1. Additional mutants were generated in a full-length Etv1 construct using the megaprimer method (49). The Gly-326–Asn-429 fragments of the Etv1 mutant derivatives were then subcloned for bacterial expression into pNIC28-Bsa4 as described and confirmed by sequencing. Expression plasmids were transformed into BL21 (DE3) Rosetta-R3 (48) or, when indicated, into Rosetta-gami^TM^ 2 (Novagen®).

##### Recombinant Protein Expression

Recombinant protein expression was induced by the addition of 0.1 mm isopropyl 1-thio-β-d-galactopyranoside to bacterial cultures grown in TB (Terrific Broth) containing 50 μg/ml kanamycin at an OD_600_ of 3.0 at 37 °C in UltraYield baffled flasks (Thomson Instrument Co, Oceanside, CA). Cultures were further incubated at 18 °C overnight. Selenomethionine-derivatized Etv1^Y329S-P427S^ expression was performed at 37 °C in M9 minimal medium, supplemented with 0.4% glucose, 2 mm MgSO_4_, 0.1 mm CaCl_2_, and 50 μg/ml kanamycin. Cells were cultured to an OD_600_ of 0.8 and 25 μg/ml selenomethionine was added, along with leucine, isoleucine, and valine to 50 μg/ml, and lysine, threonine, and phenylalanine to 100 μg/ml. Cultures were further incubated until OD_600_ of 1.2, and protein expression was induced by addition of isopropyl 1-thio-β-d-galactopyranoside and selenomethionine to final concentrations of 0.1 mm and of 75 μg/ml, respectively. Cells were harvested after further overnight incubation at 18 °C and stored at −80 °C.

##### Protein Purification

For purification of Etv1, Etv4, Etv5, and Fev constructs, ∼50 g of cell pellets were thawed and resuspended in buffer A (50 mm HEPES, pH 7.5, 500 mm NaCl, 5% glycerol, 10 mm imidazole, 0.5 mm tris(2-carboxyethyl)phosphine (TCEP)), with the addition of 1× protease inhibitor set VII (Merck, Darmstadt, Germany) and 15 units/ml Benzonase (Merck). Cells were lysed using sonication. Cell debris and nucleic acids were removed by addition of 0.15% polyethyleneimine, pH 7.5, and centrifugation at 40,000 × *g* for 1 h at 4 °C. Clarified lysates were applied to a 3-ml Ni^2+^-iminodiacetic acid-immobilized metal ion affinity chromatography gravity flow column (Generon, Maidenhead, UK), washed with 20 column volumes (CV) of buffer A, followed by 20 CV of wash buffer (buffer A with 30 mm imidazole). Fractions were eluted with 5× 2-CV aliquots of buffer A containing 300 mm imidazole and analyzed by SDS-PAGE, and relevant fractions pooled and cleaved with His_6_-tagged TEV protease (1:20 mass ratio) overnight at 8 °C. Imidazole was removed by concurrent dialysis during cleavage, using a 3.5-kDa MWCO snakeskin membrane (Thermo Fisher Scientific, Rockford, IL) in buffer B (20 mm HEPES, pH 7.5, 500 mm NaCl, 5% glycerol, 0.5 mm TCEP). TEV protease was removed from dialyzed proteins using Ni-IDA immobilized metal ion affinity chromatography (2-ml CV) and washed with an imidazole gradient in 20 mm steps to 100 mm in buffer B, and cleaved protein was pooled and concentrated with a 3-kDa MWCO centrifugal concentrators (Vivaproducts, Littleton, MA). Final separation was by size exclusion chromatography, using a HiLoad 16/60 Superdex S75 or 200 column equilibrated in buffer B, and run at 1.2 ml/min in buffer B. Protein identity was confirmed by LC/ESI-TOF mass spectrometry. Protein concentrations were calculated from *A*_280_ (Nanodrop) using the calculated molecular mass and extinction coefficients. The scheme was identical for purification of disulfide-linked proteins but with the omission of TCEP from all buffers.

##### Crystallization and Structural Determination

The purified Ets domains of Fev and Etv1 were crystallized with double-stranded DNA as follows. Oligonucleotides for co-crystallization were synthesized and used without further purification (Eurofins MWG Operon, Ebersberg, Germany). The oligonucleotides (5′-ACCGGAAGTG-3′) and (5′-CACTTCCGGT-3′), with the Ets core recognition sequence underlined, were annealed by mixing 450 μm of each oligonucleotide in 10 mm Tris, pH 7.5, 50 mm NaCl, heating to 95 °C for 5 min, and cooling to 21 °C slowly in a heating block. Frozen Etv1^Y329S-P427S^ or Fev was thawed rapidly, and aggregates were removed by microcentrifugation at 14,000 × *g* for 10 min at 4 °C. To form DNA complexes, proteins were mixed with annealed oligonucleotides in a 1:1.1 molar ratio in a buffer consisting of 10 mm HEPES, 166 mm NaCl, 5% glycerol, and 0.5 mm TCEP at protein concentrations of 5.3 and 6 mg/ml, respectively, and incubated on ice for ∼30 min. The protein/DNA mixtures were then concentrated by ultrafiltration using a 3000-Da MWCO centrifugal concentrator to an estimated protein concentration of 12.5 and 16 mg/ml for Etv1-DNA and Fev-DNA, respectively. For crystallization of the Etv4 and Etv5 DNA complexes, the same procedure was repeated but using the oligonucleotides (5′-ACCGGAAGTG-3′) and (5′ACTTCCGGTC3′). In all cases, sitting drop vapor diffusion crystallization trials were set up with a Mosquito (TTP Labtech) crystallization robot. Crystals were obtained in the following conditions: Etv1 (16 mg/ml, 20 °C), 2.5 m sodium formate; Etv-1 DNA (12 mg/ml, 4 °C), 28% PEG smear low molecular weight, 0.2 m sodium chloride, 0.1 m Tris, pH 8.5, 5% glycerol; SeMet-Etv1-DNA (15 mg/ml, 4 °C), 20% PEG 3350, 0.2 m potassium citrate; Etv4 (16 mg/ml, 20 °C), 0.8 m sodium citrate tribasic, 0.1 m cacodylate, pH 6.5; Etv4-DNA (10 mg/ml, 20 °C), 20% PEG 3350, 0.2 m magnesium chloride, 0.1 m BisTris, pH 5.5; Etv5-DNA (8 mg/ml, 20 °C), 40% PEG 300, 0.2 m calcium acetate, 0.1 m cacodylate, pH 6.0; Fev (18 mg/ml, 4 °C), 0.8 m sodium citrate tribasic, 0.1 m cacodylate, pH 6.5; Fev-DNA (16 mg/ml, 20 °C), 25% PEG 3350, 0.1 m BisTris, pH 5.5.

Crystals were cryo-protected by transferring the crystal to a solution of mother liquor supplemented with 25% ethylene glycol and flash-cooling in liquid nitrogen. Datasets were collected for all crystals at Diamond Light source beamlines I04 (Etv1, Etv1-DNA, and Fev-DNA), I02 (Fev), I24 (Etv4-DNA), and I03 (Etv4, Etv5-DNA and selenomethionine Etv1-DNA). Diffraction data were processed with the programs autoPROC (Etv1 and Etv1-DNA) (50), MOSFLM (Fev) (51), and XDS (Etv4, Etv4-DNA, Etv5-DNA and Fev-DNA) (52). The structures of Etv1, Etv4, Etv4-DNA, Etv5-DNA, Fev, and Fev-DNA were solved by molecular replacement using the program PHASER (53). Initial attempts to solve the Etv1-DNA complex by molecular replacement were unsuccessful, and for this reason, the selenomethionine-derivatized protein was produced, and SAD data were collected close to the selenium peak wavelength (0.976 Å). Selenium atom positions were located with the program SHELX (54) and refined using the program SHARP (55). Model building and manipulation were performed in COOT (56), and the structures were refined using autoBUSTER (Global Phasing) (Etv1, Etv1-DNA, and Fev), REFMAC (Etv4) (57), and PHENIX REFINE (Etv4-DNA, Etv5-DNA and Fev-DNA) (58). A summary of the crystallization conditions, data processing, phasing, and refinement statistics for all datasets are shown in Tables 1 and 2.

**TABLE 1:**
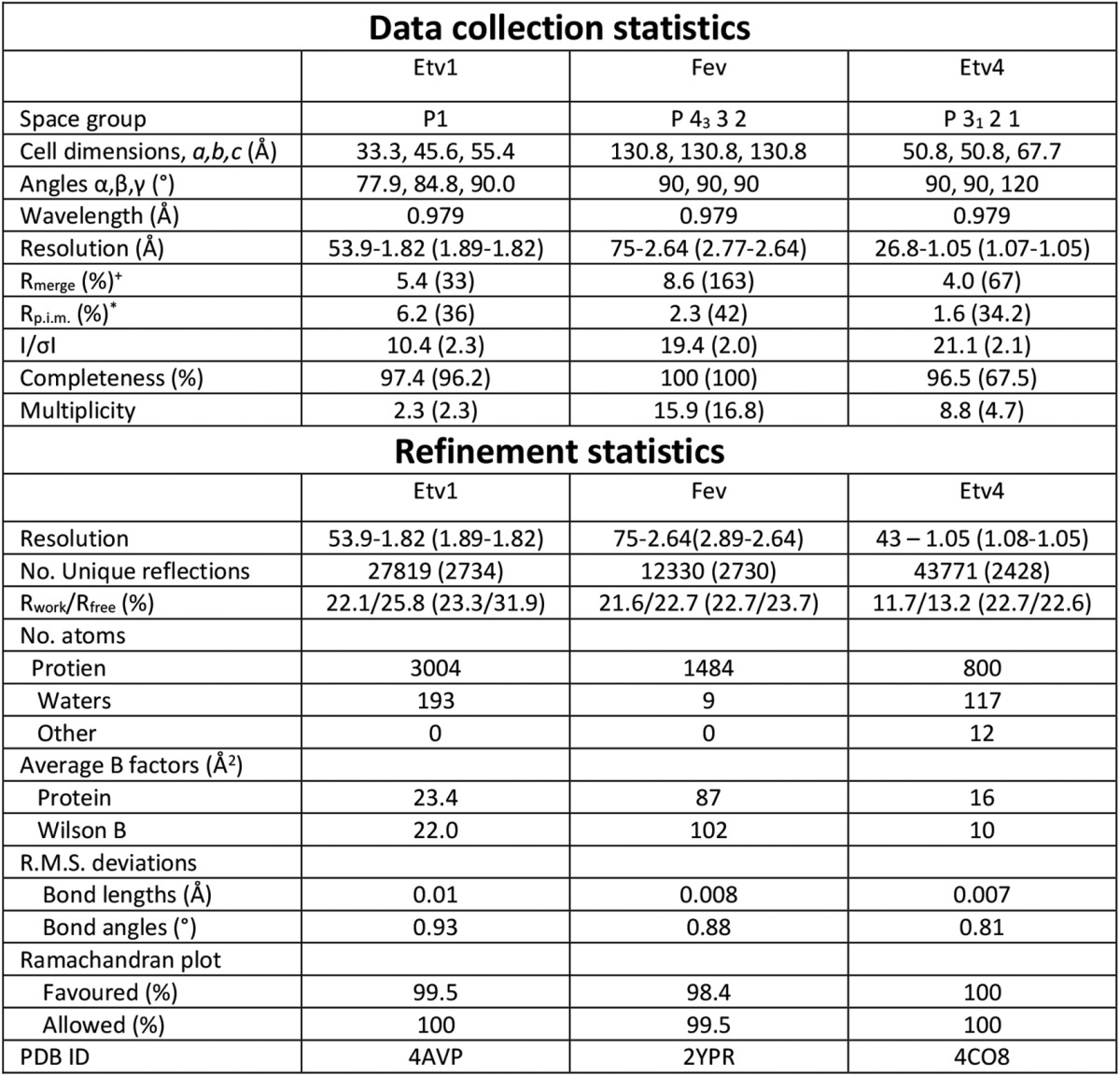
**Data collection and refinement statistics for DNA free structures** Values in parentheses are for the highest resolution shell.

^+^*R*_merge_ = Σ_*hkl*_Σ_*i*_|*I_i_* − I*_m_*|/Σ*_hkl_*Σ*_i_I_i_*, where *I_i_* and *I_m_* are the observed intensity and mean intensity of related reflections, respectively.

*R_p.i.m._ = Σ*_hkl_*√(1/n − 1) Σ_*i*_*n* = 1__|*I_i_* − *I_m_*|/Σ*_hkl_*Σ*_i_ I_i_*.

**TABLE 2:**
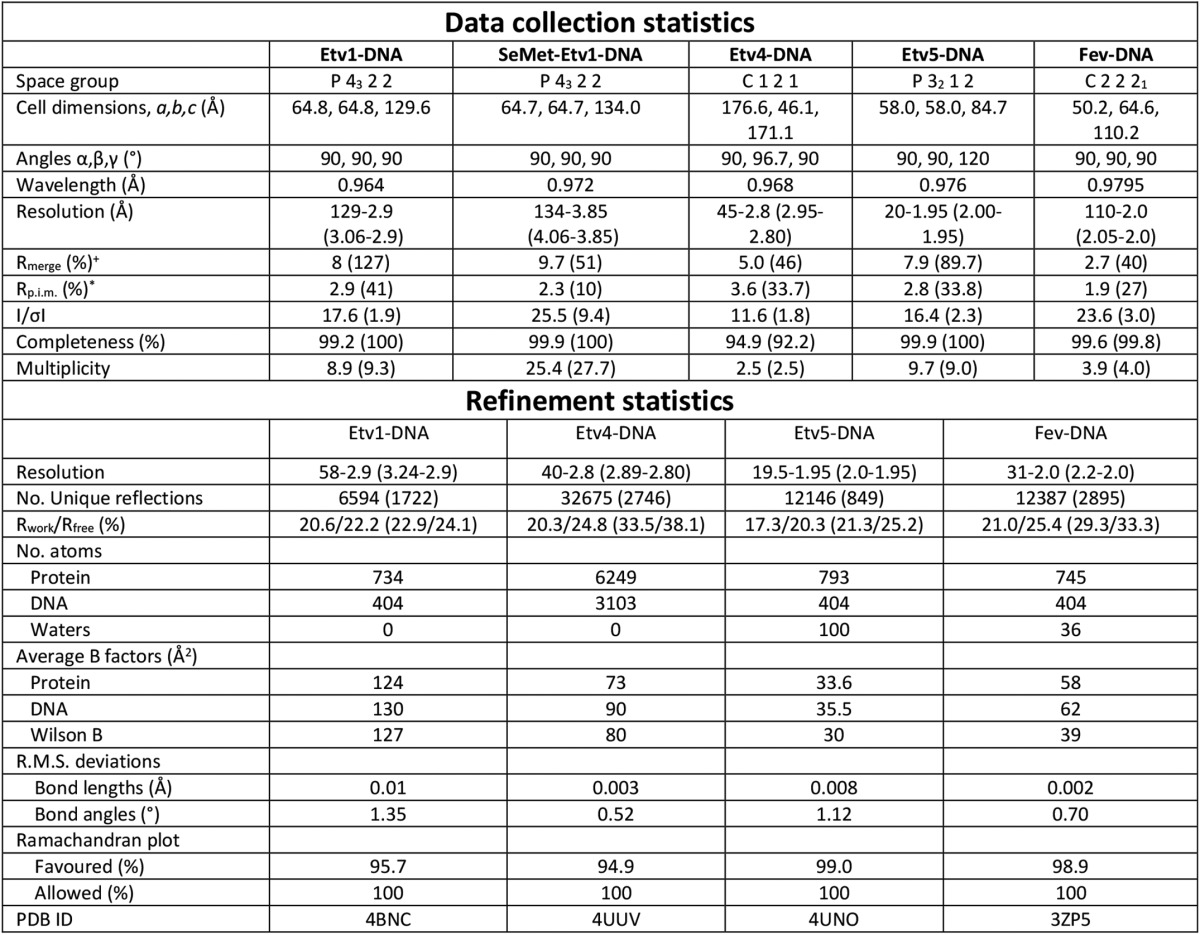
**Data collection and refinement statistics for DNA complexes** Values in parentheses are for the highest resolution shell.

^+^*R*_merge_ = Σ_*hkl*_Σ_*i*_|*I_i_* − I*_m_*|/Σ*_hkl_*Σ*_i_I_i_*, where *I_i_* and *I_m_* are the observed intensity and mean intensity of related reflections, respectively.

*R_p.i.m._ = Σ*_hkl_*√(1/n − 1) Σ_*i*_*n* = 1__|*I_i_* − *I_m_*|/Σ*_hkl_*Σ*_i_ I_i_*.

##### LC/ESI-TOF Mass Spectrometry of Intact Proteins

30-μl protein samples at 0.02 mg/ml in 0.1% formic acid were injected onto a 4.6 × 50 mm Zorbax 5-μm 300SB-C3 column and resolved by reversed-phase chromatography at 40 °C. The solvent system was 0.1% formic acid in double distilled H_2_O (buffer A) and 0.1% formic acid in methanol (buffer B), with 1 min at 5% buffer B and then a linear gradient of 5–95% buffer B over 6 min at 0.5 ml/min. Protein intact mass was determined using an MSD-TOF electrospray ionization orthogonal time-of-flight mass spectrometer (Agilent Technologies, Palo Alto, CA) operated in positive ion mode.

##### In Vitro Protein Phosphorylation

Phosphorylation of Etv1 Ser-334 by protein kinase A was performed *in vitro* using 2.5 units of bovine heart PKA catalytic subunit (P2645, Sigma) with 40 μg of Etv1, in modified buffer B (20 mm HEPES, pH 7.5, 500 mm NaCl, 5% glycerol, 1 mm ATP, 10 mm MgCl_2_, and 5 mm dithiothreitol). Reactions were performed for 4 h at room temperature and quenched by the addition of 50 mm EDTA, followed by buffer exchange into buffer B with Micro Bio-Spin® P-6 columns (Bio-Rad) and confirmation of phosphorylation by ESI-TOF mass spectrometry of the intact protein.

##### Electrophoretic Mobility Shift Assays

Equilibrium binding constants (*K_d_*) of Ets constructs were estimated by EMSA. Unless otherwise specified, the dsDNA probe contained the single consensus Ets-binding site used in crystallization (underlined), with oligonucleotides Etv1ALF (5′-ATCTCACCGGAAGTGTAGCA-3′) and Etv1ALR (5′-TGCTACACTTCCGGTGAGAT-3′). Substrates were prepared by ^32^P-end-labeling one oligonucleotide, annealing to the complementary strand in 10 mm Tris-Cl, pH 7.5, 50 mm NaCl, prior to purification with Micro Bio-Spin® P-6 columns. Proteins were generally titrated from 10^−14^ to 10^−6^ m. The DNA probe was used at 2 nm for the initial estimations of *K_d_* (termed *K*_*d*_^app1^ in Table 3). As the dissociation constants of wild-type Etv1 were estimated to be subnanomolar, we repeated some of the measurements with a DNA probe concentration of 0.1 nm to maintain an excess of protein (results listed as *K*_*d*_^app2^ in Table 3). As seen in Table 3, the effects of mutations are qualitatively similar at both DNA concentrations. EMSA buffer is composed of 50 mm Tris-HCl, pH 7.5, 50 mm NaCl, 2 mm MgCl_2_, 0.01% Tween® 20, and 5% glycerol. Proteins were reduced with 10 mm TCEP prior to assay unless otherwise specified. Reactions were performed for 1 h at room temperature, prior to mixing with loading dye to 0.25%, and resolved by 10% native PAGE at 150 V for 1 h at room temperature. Gels were analyzed using phosphorimaging, and the apparent *K_d_* value was estimated from plots by nonlinear regression with a least squares fit, using a specific one-site binding model with Hill slope (Prism, GraphPad, San Diego). Other EMSA substrates are described in the respective figure legends.

**TABLE 3:** **DNA binding affinity of Ets domain constructs (Etv1 unless specified)** NA indicates undetectable binding or below threshold for regression analysis. *K*_*d*_^app1^ indicates apparent *K_d_* value measured with a 2 nm DNA probe. *K*_*d*_^app2^ indicates apparent *K_d_* value measured with a 0.1 nm DNA probe.

Construct (Etv1 unless specified otherwise)	Description/mutation	*K*_*d*_^app1^ (×10^−10^ m)*^a^*	*K*_*d*_^app1^-fold inhibition*^b^*	*K*_*d*_^app2^ (× 10^−10^ m)*^a^*	*K*_*d*_^app2^-fold inhibition*^b^*
WT	Wild type Ets domain	2.7 ± 0.28	1	5.9 ± 1.5	1
Y329S-P427S	PCR mutant Ets domain	2.5 ± 0.26	0.9	3.1 ± 0.6	0.5
K379A	DNA backbone contact	NA	NA		
D387A	DNA base contact	1.7 ± 0.2	0.6	1.9 ± 0.3	0.3
R391A	DNA base contact	118 ± 14.8	43.7	86.0 ± 16.8	14.6
R394A	DNA base contact	2757 ± 627	1021	ND	ND
Y395F	DNA base contact	2.3 ± 0.5	0.9	13.5 ± 10.5	2.3
Y396F	DNA backbone contact	26390 ± 9349	9774.1		
Y397F	DNA backbone contact	NA	NA		
K404A	DNA backbone contact	NA	NA		
S334E	Phosphoserine mimic	8.9 ± 3.2	3.3	9.4 ± 5.1	1.6
WT (phosphorylated)	PKA-phosphorylated Ser-334	521 ± 455	193		
C416S	Abrogation of disulfides	4.6 ± 0.6	1.7	2.6 ± 0.6	0.4
WT dimer	Disulfide-linked dimeric WT ETV1	195 ± 148	72.2	802 ± 164	136
WT dimer (TCEP reduced)	Dimeric WT ETV1, reduced	11.2 ± 2.6	4.1	2.6 ± 0.4	0.4
WT dimer (9:1 GSH:GSSG)	Dimeric WT ETV1, reduced			171 ± 23	29
WT dimer (1:9 GSH:GSSG)	Dimeric WT ETV1, reduced			272 ± 30	47
Etv4 WT (reduced)	Wild type Ets domain, reduced			3.4 ± 0.66	1
Etv4 WT (oxidized)	Disulfide-linked dimeric WT ETV4			148 ^c^ ± 35	44
Etv5 WT (reduced)	Wild type Ets, reduced			11.6 ± 1.1	1
Etv5 WT (oxidized)	Disulfide-linked dimeric WT ETV5			790 ± 610*^c^*	68

*^a^ K_d_* ± S.E.

*^b^* Fold increase in *K_d_* value compared with the corresponding wild type.

*^c^* Estimated, due to binding at regression analysis threshold.

## Results

### 

#### 

##### DNA-free Structures of the Ets Domains of Etv1, Etv4, and Fev

The full-length sequences of Etv1, Etv4, Etv5, and Fev are predicted to include disordered regions, which often hinder crystallization. We have generated several expression constructs, including the full-length and truncated versions of each protein. Crystals were obtained from constructs that contained the Ets domain, Etv1 (encompassing amino acids 326–429), Etv4 (encompassing amino acids 338–470), and Fev (encompassing amino acids 42–141). The construct of Etv1 included two inadvertent mutations that resulted from primer impurities as follows: Y329S at the N terminus, and P427S at the C terminus; this construct is denoted Etv1^Y329S-P427S^. Our attempts to crystallize the corresponding construct with the wild-type sequence failed, although the mutated residues are in disordered regions or at the end of the ordered region of the crystal structures of Etv1, so they are unlikely to have any bearing on the structural and functional analyses. We have also attempted to crystallize Etv5 using a similar construct strategy to that defined above (Etv5 construct encompassing amino acids 365–462), but despite obtaining protein of similar purity and yield as the other Etv constructs, we were unable to grow crystals of Etv5 in the absence of DNA.

**FIGURE 1.**
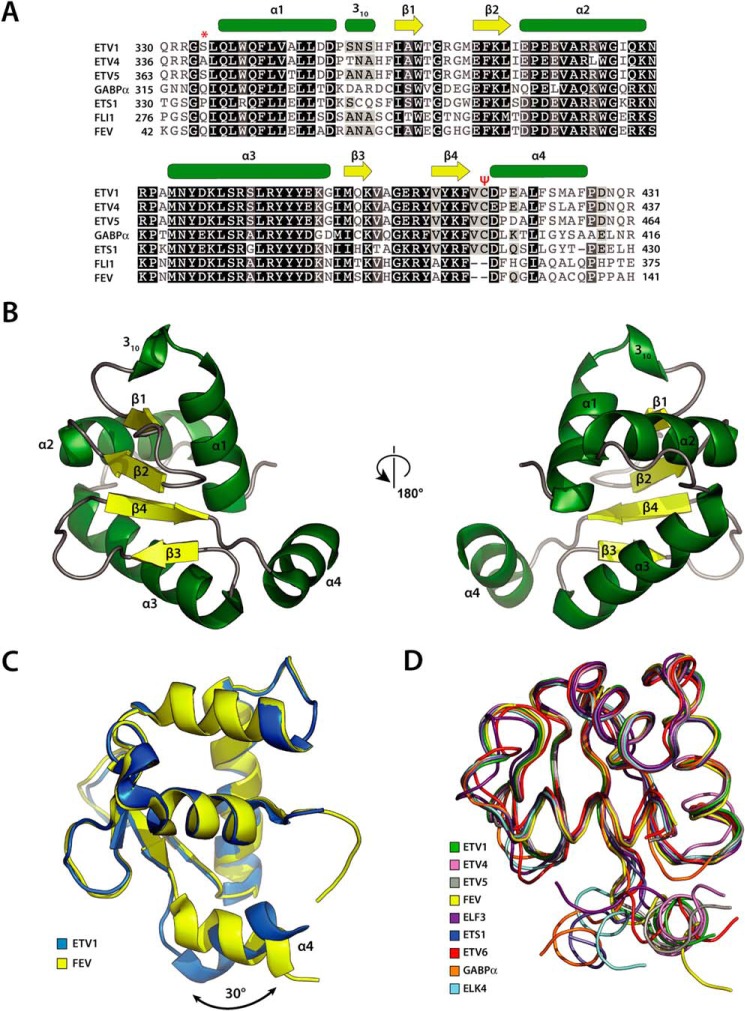
**Etv1 and Fev Ets domain topology and sequence alignment.** *A,* sequence alignment of class I Ets domains. Sequences were Etv1 (AAD29877), Etv4 (AAH16623), Etv5 (CAG33048), GABPα (NP_001184226), ETS1 (CAG47050), FLI1 (AAH10115), and Fev (NP_059991). Secondary structural assignment is for Etv1 (PDB code 4AVP), containing additional flanking residues where appropriate. *Green,* helix (α or 3_10_); *yellow,* β-strand; *, serine phosphorylated by protein kinase A; ψ, conserved cysteine involved in disulfide bond formation in Etv1, -4, and -5. *B,* overall cartoon representation of Etv1 Ets domain. *Green*, helix (α or 3_10_); *yellow*, β-strand. *C,* superimposition of Etv1 (PDB code 4AVP, *blue*) and Fev (PDB code 2YPR, *yellow*). *D,* ribbon superimposition showing the variance in the positions of α4. Structures used were Etv1 (PDB code 4AVP), Fev (PDB code 2YPR), ELF3 (PDB code 3JTG), ETS1 (PDB code 3MFK), Etv6 (PDB code 2LF7), GABPα (PDB code 1AWC), and ELK4 (PDB code 1BC8).

Crystals of Etv1^Y329S-P427S^ diffracted to 1.82 Å and contained four copies of Etv1 in the asymmetric unit (4AVP, Tables 1 and 2). Electron density is observed for residues Ser-334 to Phe-426 (thus excluding the Y329S and P427S mutations) with most side chains visible. Etv4 crystals diffracted to 1.05 Å and contained a single copy of Etv4 in the asymmetric unit (4CO8, Tables 1 and 2). Fev crystals diffracted to 2.64 Å, with two copies of Fev per asymmetric unit (2YPR, Tables 1 and 2). Ets domains are highly similar in sequence (Fig. 1*A*), with Etv1, Etv4, and Etv5 all sharing over 90% sequence identities, whereas Fev is slightly more distantly related (∼65% identity).

In the structural descriptions that follow, the protein residues are numbered according to their position in Etv1; a list of the corresponding residues in Etv4 and Etv5 and Fev is provided in Table 4.

**TABLE 4:** **Numbering of equivalent residues in Etv1, -4, and -5 and Fev**

Etv1	Etv4	Etv5	Fev
Gln-336	Gln-342	Gln-369	Gln-48
Leu-337	Leu-343	Leu-370	Leu-49
Trp-375	Trp-381	Trp-408	Trp-87
Lys-379	Lys-385	Lys-412	Lys-91
Asp-387	Asp-393	Asp-420	Asp-99
Lys-388	Lys-394	Lys-421	Lys-100
Arg-391	Arg-397	Arg-424	Arg-103
Ser-392	Ser-398	Ser-425	Ala-104
Arg-394	Arg-400	Arg-427	Arg-106
Tyr-395	Tyr-401	Tyr-428	Tyr-107
Tyr-396	Tyr-402	Tyr-429	Tyr-108
Tyr-397	Tyr-403	Tyr-430	Tyr-109
Lys-399	Lys-405	Lys-432	Lys-111
Lys-404	Lys-410	Lys-437	Lys-116
Arg-409	Arg-415	Arg-442	Arg-121
Tyr-410	Tyr-416	Tyr-443	Tyr-122
Cys-416	Cys-422	Cys-449	Phe-F126

Etv1, Etv4, and Fev display the typical fold of the Ets domain (Fig. 1, *B* and *C*), which contains three α-helices (α1–3) flanking a four-stranded β-sheet, with an additional C-terminal helix (α4). The structures are highly similar (typical root mean square deviation of 1.0 Å over ∼90 residues), with the main difference being a two-amino acid deletion in Fev (Fig. 1*A*), linked to a shift of 30° in the orientation of the C-terminal α4 helix in the Fev structure compared with the other structures (Fig. 1*C*). Superposition of the main chains of other Ets domain structures reveals considerable heterogeneity in the region corresponding to helix α4 (Fig. 1*D*). This region was not expected to be helical from proline-scanning mutagenesis (59) and was not structured in the NMR ensemble of the closest structural homologue of Fev, Fli1 (31).

##### Crystal Structure of the Etv1, Etv4, Etv5, and Fev DNA Complexes

**FIGURE 2.**
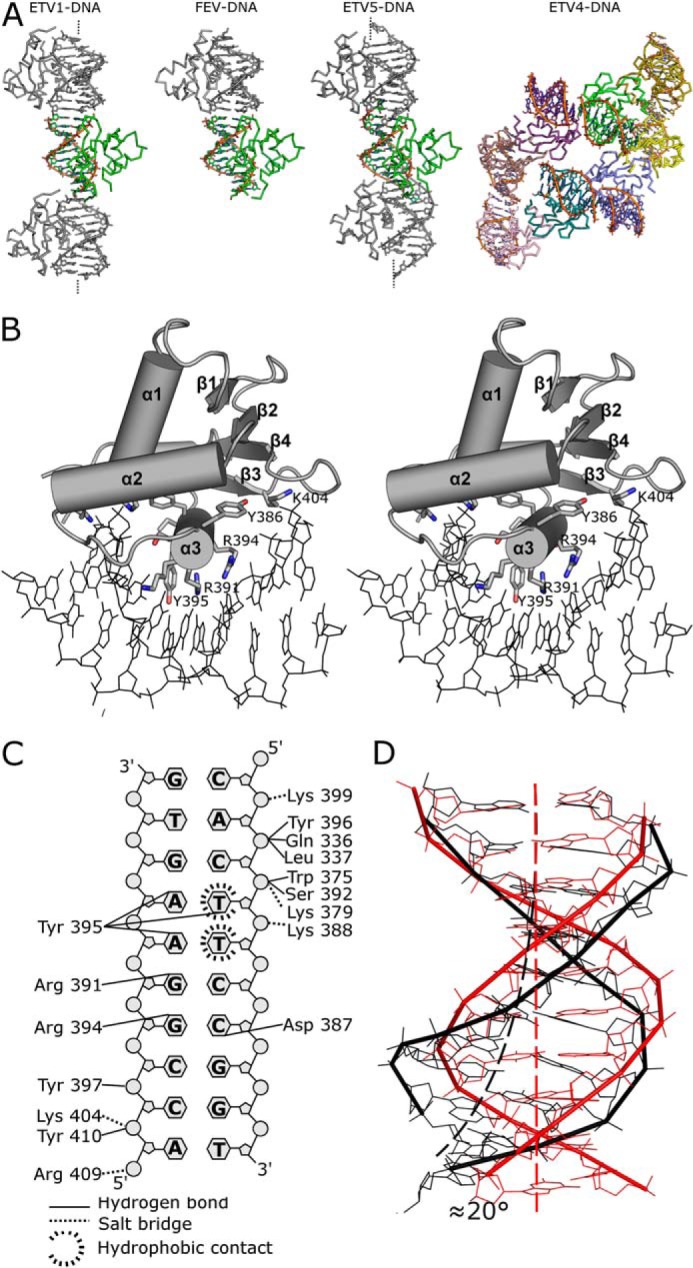
**Interactions of Etv1 with DNA.** *A,* contacts formed by DNA complex crystals. The DNA molecules in the DNA complex crystals associate via blunt (Etv1 and Fev) or sticky ends (Etv4 and Etv5) with neighboring molecules in the crystal. The contents of the asymmetric unit are colored by chain with symmetry-related molecules colored *gray*. DNA fibers ending in *dashed lines* form a continuous helix running the entire length of the crystal. *B,* stereo view of the interaction between Etv1 and DNA. Etv1 is shown in cartoon representation with secondary structure elements labeled; residues forming close contacts with the DNA are shown as *sticks,* and the DNA is shown in the *line* representation. The DNA can be seen to undergo a significant widening of the major groove to accommodate extensive interactions with residues from the recognition helix α3. *C,* schematic view of the residues involved in the DNA protein interface of both Etv1 (shown on the *left*) and Fev (shown on the *right*). Hydrogen bonds are depicted as *solid black lines*, and salt bridges are represented as *dashes. D,* comparison of the DNA from the Etv1 DNA complex (*black*) with canonical B form DNA of similar length (*red*). The DNA can be seen to undergo a significant widening of the major groove and bending of ∼20° toward the protein interface.

Both Etv1 and Fev DNA complexes were crystallized using a single 10-base pair DNA duplex containing the class I consensus EBS sequence. Etv4 and Etv5 DNA complexes were crystallized using oligonucleotide duplexes that had nine complementary base pairs (containing the same consensus sequence) with a single overhanging nucleotide on each end, which makes favorable interactions with neighboring DNA molecules (Fig. 2*A*). Etv1-DNA crystals diffracted to 2.9 Å and contained a single copy of Etv1 and a single DNA duplex in the asymmetric unit (4BNC, Table 2). Etv4-DNA crystals diffracted to 2.8 Å resolution and contained eight Etv4 molecules and eight DNA duplexes in the asymmetric unit (4UUV, Table 2), with the only significant differences between the various chains in the asymmetric unit being the chemical environment of the DNA chains. Etv5-DNA crystals diffracted to 1.95 Å and contained a single copy of Etv5, a single calcium ion (bound close to the N terminus of α4), and a single DNA duplex in the asymmetric unit (4UN0, Tables 1 and 2). Fev-DNA crystals diffracted to 2.64 Å, with two copies of Fev per asymmetric unit (2YPR, Tables 1 and 2). In all the DNA complex crystals, base pair stacking interactions between neighboring molecules allow the DNA to form a pseudo-contiguous helix that, in some cases, runs the entire length of the crystal (Fig. 2*A*).

In all four DNA complexes, the recognition helix (α3) inserts deep into the major groove and provides multiple contacts with the base pairs of the core GGAA sequence, as well as with the phosphate backbone (Fig. 2, *B* and *C*). Additional contacts to the DNA backbone are provided by the C terminus of α2, the α2-α3 loop, β3, and the β3-β4 loop. The proteins contact the DNA over a span of 9 bp, and the interface accounts for the burying of ∼9% (500 Å^2^) of the total solvent-accessible surface area. To accommodate these extensive interfaces, the DNA becomes slightly distorted from the canonical B form, exhibiting smooth bending of ∼20° toward the protein with significant widening of the major groove (∼20.5 Å at widest point) in the vicinity of the recognition helix and narrowing of the minor groove downstream (∼9.3 Å at narrowest point) (Fig. 2*D*). The bending of the DNA can be explained by the large number of contacts made to the phosphodiester backbone, many of which are charged in nature possibly introducing bending through asymmetric phosphate neutralization (60). Specifically in Etv1, hydrogen bonds to phosphate oxygens are formed with the side chains of residues Gln-336, Trp-375, Tyr-386, Ser-392, and Tyr-396, whereas salt bridges are formed with Lys-379, Lys-388, Lys-399, and Lys-404 (Fig. 2*C*). Two additional charged residues, Arg-381 and Arg-409, appear to be in position with the potential to interact with the phosphodiester backbone; the former occupies a position between the two DNA strands in the region of the minor groove narrowing, and the latter could potentially form an additional salt bridge in longer DNA sequences. All of the equivalent interactions are preserved in Etv4, Etv5, and Fev with the exception of Arg-381 and Ser-392, which are replaced by Lys-93 and Ala-104, respectively, in Fev.

##### Recognition of Specific DNA Substrates by Etv1 and Fev

**FIGURE 3.**
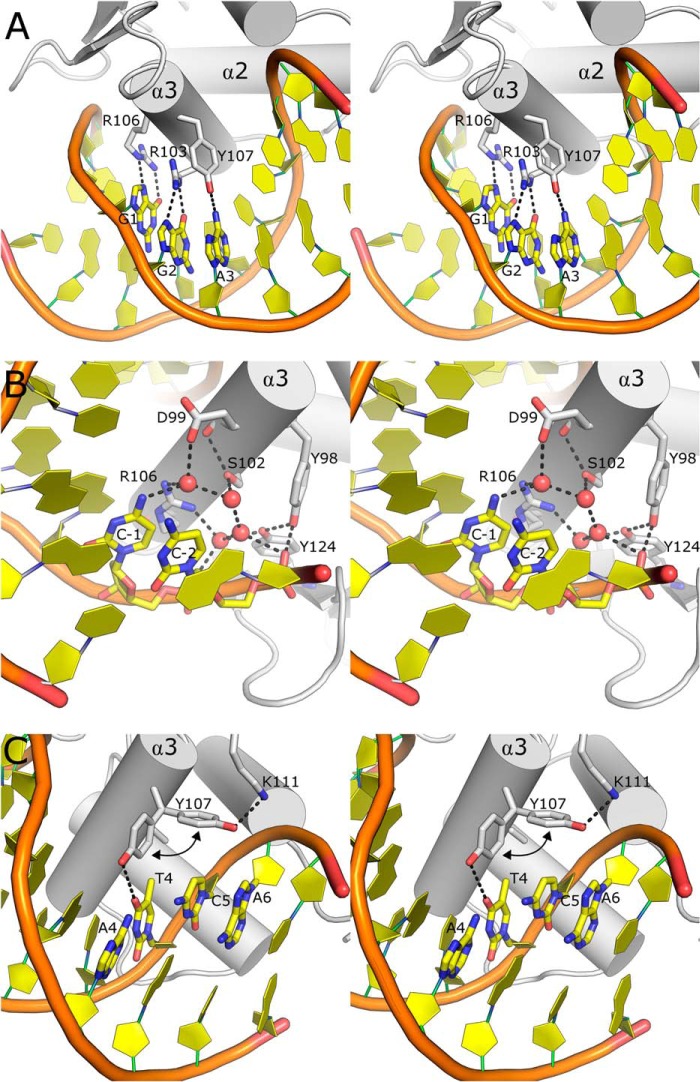
**Molecular basis of DNA sequence recognition.** *A,* stereo view of the sequence-specific protein DNA interactions between Fev and DNA that facilitate the recognition of the core GGA motif. Key residues and nucleobases are shown in the *stick* representation, and hydrogen bonds are shown as *dotted lines. B,* recognition of features upstream of the GGA core is achieved via a highly coordinated network of water molecules that are conserved among other class I ETS domains. *C,* recognition of DNA sequence features downstream of the core GGA motif by the dual conformation of Tyr-107, which appears to be able to form both polar and nonpolar interactions with the thymine at position 4, and in its alternative conformation it forms van der Waals interactions with nucleobases at the +4 and +5 positions on the complementary strand from the GGA core.

The Ets family of transcription factors display sequence-specific DNA recognition for nine residues, which we have numbered −3 to +6 around the invariant 5′-GGA-3′ core. This core GGA sequence is directly recognized in all Ets family members by two arginines and a tyrosine (Arg-391, Arg-394, and Tyr-395 in Etv1; Arg-103, Arg-106, and Tyr-107 in Fev), found invariably on the recognition helix (α3). The two arginine residues are both positioned with their guanidinium groups directly above the O6 and N7 of the two guanine bases and, due to the requirement for two hydrogen bond acceptors, are capable of direct recognition of guanine at positions 1 and 2 (Fig. 3*A*). Similarly, the tyrosine is in a position to accept a hydrogen bond from the N6 of the adenine residues at position 3 (Fig. 3*A*); positioning of the complementary thymine methyl group in a nonpolar environment created by the aliphatic portion of the side chains of Arg-391 (Fev-Arg-103) and Lys-388 (Fev-Lys-100) may also contribute to recognition at this position. The characteristic positioning of the two arginine side chains is likely to be further stabilized by cation-π stacking interactions, which, in the case of Ets domains, form between the guanidinium group of the arginine and the base at the −1 position, on the same strand as the hydrogen bonded partner. These types of interactions have already been identified in other Ets domain DNA complexes and may contribute to sequence specificity at this site due to the fact that arginine-guanine interactions are energetically the most favorable (61, 62).

**FIGURE 4.**
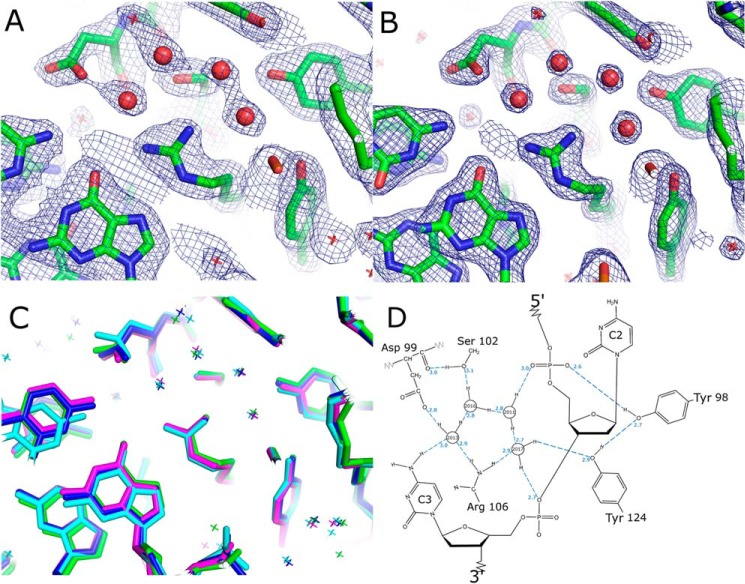
**Cluster of conserved water molecules in the protein DNA interface.** *A,* 2*F_o_* − 1*F_c_* electron d.5σ. *B,* 2*F_o_* − 1*F_c_* electron density map of the Etv5-DNA complex, contoured at 1.5σ. *C,* structural superposition of the Fev (*green*), Etv5 (*blue*), SAP-1 (PDB code 1BC8, *cyan*), and ELK1-DNA (PDB code 1DUX, *pink*) complexes showing the conserved positions of the four water molecules important for recognition of bases at the −1 and −2 positions. *D,* schematic view of the water network with hydrogen atoms shown explicitly and hydrogen bonds and distances shown in *blue*. A single arrangement of hydrogen atoms is sufficient to fulfill all of the required donor-acceptor pairs, and it includes the requirement for a hydrogen bond donor at the −1 position, explaining the observed substrate specificity at this site.

The DNA sequences recognized by different subclasses of the Ets family outside the central GGA core are more diverse (32, 33). Etv1, Etv4, Etv5, and Fev all belong to the class I subfamily of Ets domains with a preferred consensus sequence 5′-ACCGGAAGT-3′. Both Etv1 and Fev display an absolute requirement for positions +1 to +3, strong preferences at positions −1, −2, and +4, and somewhat more relaxed sequence selectivity at positions −3, +5, and +6 (32). The means by which this recognition is achieved is not currently well understood, as relatively few direct base contacts outside the core GGA motif can be seen in the current structures in the PDB (30, 63–65). From analysis of the high resolution structures of the Etv5 and Fev DNA complexes (which are high enough resolution (< 2.0 Å) to reliably locate ordered waters), a number of additional contacts can be seen, which appear to enable direct readout of additional base pairs both upstream and downstream of the GGA core. The two C-G base pairs at positions −2 and −1 lie close to a highly coordinated network of four water molecules conserved in both structures and are coordinated by polar contacts to the side chains of Asp-387 (Fev Asp-99), Ser-390 (Fev Ser-102), Arg-394 (Fev Arg-106), Tyr-412 (Fev Tyr-124), and the phosphate oxygens of the two cytosine nucleotides at positions −2 and −1. These waters are also found in conserved positions in the crystal structures of the DNA complexes of SAP-1 (64) and ELK-1 (65) and form a hydrogen bond to the N4 of the cytosine at position −1 (Fig. 3*B*, Fig. 4, *A–C*). A detailed examination of the chemical environment of these water molecules using known hydrogen bonding donors and acceptors as fixed entities (assuming normal protonation states at physiological pH) reveals a single unique arrangement that satisfies all of the interactions in the network (Fig. 4*D*). The requirement in this network for the water closest to the base at position −1 to be a hydrogen bond acceptor almost perfectly explains the observed sequence preferences at this position (32), with a strong selection of cytosine (∼75% occurrence) and the only other allowed base being adenine (∼15% occurrence), which is also able to donate a hydrogen bond, although in this case the hydrogen bonding distance would be significantly longer (≈4.0 Å). The close packing of these water molecules also appears sufficient to explain the selectivity at the −2 position (in which cytosine is the most favored base and thymine is the least frequent), due to the potential to form favorable van der Waals-type interactions with the nonpolar face of the cytosine and potentially unfavorable interactions or steric clashes with the thymine methyl C7. This mode of recognition also likely occurs at the −1 position; consequently, there would be a similar discrimination against 5-methylcytosine, which could occur in position −1 (which is part of a CpG base step). Interestingly, one high resolution crystal structure in which the positions of these waters is not conserved, the PU.1 DNA complex (66), is a member of the class III subfamily of Ets domains that are specific for EBS sequences containing adenine and guanine in the −1 and −2 positions, respectively (32). The base pairs at positions +4 and +5 downstream from the GGA motif lie close to the side chain of Tyr-395, which has the potential to accept a hydrogen bond from the N6 of the adenine at position +4, and also to form a favorable van der Waals interaction with the corresponding thymine C7 methyl.

In the Etv5 and Fev DNA complex structures, the corresponding residue (Etv5-Tyr-428 and Fev-Tyr-107, respectively) can be seen to occupy two conformations, one in which the two interactions detailed above are conserved, and an additional conformation where the side chain hydroxyl lies close to Etv5-Lys-432/Fev-Lys-111 and provides a large contact surface, with the potential for favorable van der Waals interactions to bases at positions +5 and +6 on the complementary strand (Fig. 3*C*). As discussed below, mutation of the corresponding tyrosine in Etv1 (Tyr-395) to phenylalanine had little effect on DNA binding affinity, suggesting that this van der Waals interaction is the most significant contribution of this residue to binding energy. Although these interactions may not be sufficient to determine the absolute sequence preferences at these positions, the experimentally derived preferences are more relaxed, with a tolerance for thymine at +4, adenine at +5, and cytosine at +6 (32). Both the importance to DNA recognition and the dynamic nature of this residue have already been demonstrated in a comparative structural study of the DNA binding properties of the Elk-1 and Sap-1 Ets domains (65) in which a salt bridge, established between the nearby Lys (equivalent to Fev-Lys-111) and a neighboring Asp residue (equivalent to Fev-Asp-110), was thought to stabilize the tyrosine in its alternative position. The finding that both conformations of this residue appear to occur together indicates that the dynamic nature of the side chains of the recognition helix may be even more important for DNA recognition than previously thought.

##### Order-Disorder Transitions upon DNA Binding

**FIGURE 5.**
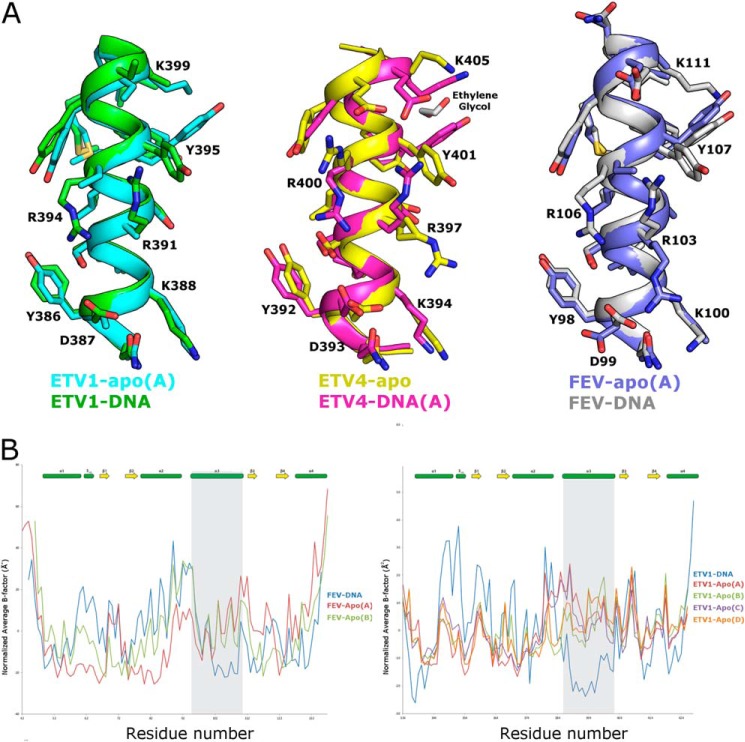
**Structural transitions on DNA binding in Fev and Etv1.** *A,* comparison of the apo- and DNA-bound forms of Etv1 (shown on the *left*), Etv4 (*center*), and Fev (shown on the *right*) reveals significant disorder-order transitions and rotamer movements within the recognition helix that accompany DNA binding. *B,* comparison of temperature factors of apo- and DNA-bound forms. The average *B*-factor (normalized so that the mean value is equal to zero) is plotted as a function of residue number for Fev (*left panel*) and Etv1 (*right panel*), with secondary structural elements shown for reference. Residues belonging to the recognition helix α3 are highlighted by a *gray background* and can be seen in both cases to transition from relatively high *B*-factors to among the lowest *B*-factors in the entire structure. These transitions, however, were not seen in a comparison of Etv4 apo- and DNA-bound crystals, probably due to the extensive crystal contacts formed by residues in this helix in the apo-crystals, which consequently diffract to 1.05 Å resolution.

A comparison of the structures of Etv1, Etv4, and Fev in the presence and absence of DNA allows us to identify conformational changes that may occur upon DNA binding. In both cases, the overall structure is very well conserved (root mean square deviation of ∼1 Å), with only minor movements of both the N and C termini and the β3-β4 loop, which appear to move away from the DNA in the DNA complexes of both Etv1 and Fev, avoiding potential steric overlaps that can be seen to occur following structural superposition. On an individual residue level, the most striking transitions can be seen to occur in the recognition helix α3, where in Etv1 Asp-387, Arg-391, and Arg-394 can be seen to become ordered upon DNA binding (Fig. 5, *A*, and *B*) and Tyr-395, which can be seen to switch between the two alternative rotamers found to be important for recognition of bases downstream of the GGA motif in the Fev-DNA complex. Similar transitions occur in the Fev structure where Asp-99, Arg-103 and Tyr-107 adopt different rotamers, and the disordered Arg-106 and Lys-111 become ordered upon DNA binding. In Etv4, in addition to three residues Arg-397, Arg-400, and Tyr-401 adopting different rotamers, the C-terminal end of the recognition helix switches from a 3_10_ hydrogen bonding pattern (which is the form adopted in all the other ETS structures) to a more canonical α-helical hydrogen bonding pattern, although in this case the difference may be due to the fact an ethylene glycol molecule is bound in the vicinity (Fig. 5*A*). The dynamic nature of the residues within the recognition helix is likely to be an important factor for Ets domain recognition, and it indicates possible means whereby Ets domains may scan along the DNA duplex in search for high affinity sites following binding to nonspecific sites, in a similar manner to that observed for the *lac* repressor DNA complex (67).

##### Biochemical Analysis of Etv1 DNA Binding

**FIGURE 6.**
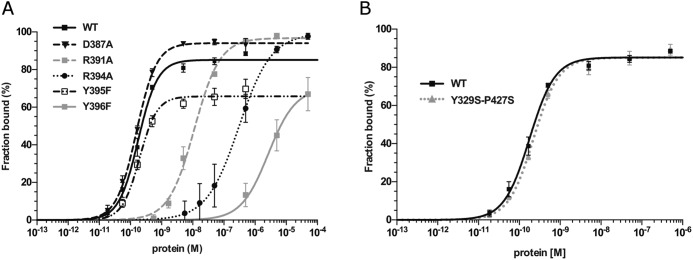
**Mutational analysis of Etv1 DNA binding.** *A,* DNA binding isotherms of mutated Etv1 proteins derived from EMSAs using the standard DNA probe at 2 nm. *Error bars* are plotted as ± S.E. of replicates. *B,* comparison of the DNA binding to WT and the primer-induced mutant Etv1 (Y329S/P427S) used for crystallization.

To assess the contribution of individual residues to DNA binding in the solution we have, using Etv1 as a model system, mutated several residues individually to alanine or (in the case of tyrosine residues) to phenylalanine. Isolated Ets domains containing each of the mutations were compared in EMSAs to wild-type Etv1 (Fig. 6*A* and Table 3). We also compared the Etv-1 construct used for crystallization, which contains two primer-induced mutations, against wild-type Etv1 and found no significant difference in DNA binding (Fig. 6*B*). Somewhat surprisingly, mutants in residues involved in salt bridges to the DNA backbone (Lys-379 and Lys-404) exhibited no detectable DNA binding. Similarly, mutants of the tyrosine residues forming hydrogen bonds to the DNA backbone (Tyr-396 and Tyr-397) to either phenylalanine or to alanine also resulted in significant (>1000-fold) reduction in binding affinity to DNA.

Mutations in individual residues involved in nucleobase interactions affected DNA binding to a lesser extent. Mutants of the invariant Arg-391 and Arg-394 bound DNA 50- and 1300-fold less than the wild type, respectively. Interestingly, mutation of Tyr-395 to phenylalanine did not affect the *K_d_* values, but maximal DNA binding at saturation was only 60% of the total probe; this may indicate a rapid dissociation (high *k*_off_) of the complexes. Uniquely among the residues tested, a D387A mutation had no effect on DNA binding; it is possible that this residue contributes little to the free energy of binding to the cognate DNA sequence but may be crucial for selectivity (see below). Overall, the mutational analysis demonstrates that the residues interacting with the DNA backbone in the crystal structure are the major contributors to the binding affinity in solution, although the interactions with the bases add a significant but lower energetic contribution.

##### Biochemical Analysis of Etv1 Binding to Methylated DNA

**FIGURE 7.**
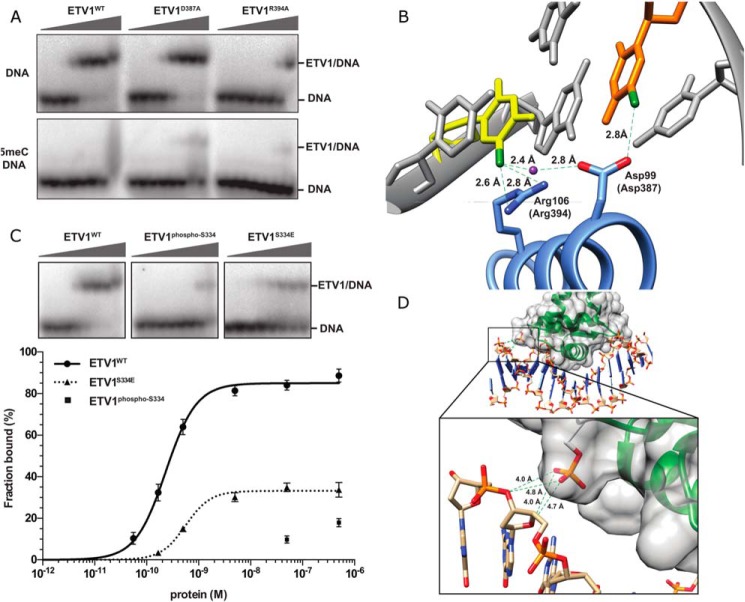
**Effects of methylation and phosphorylation on Etv1 DNA binding.** *A,* representative EMSA analysis of three Etv1 proteins (wild type, D387A, and R394A) titrated against normal (*upper panel*) and 5meC-modified DNA (Etv1ALF-5meC/Etv1ALR-5meC, *lower panel*). Gradients represent increasing protein concentrations, and unbound DNA substrate and complexes are marked. *B,* model of potential interactions with 5-methylated cytosine, based on the Fev-dsDNA structure (PDB code 3ZP5) with methyl groups on the methylated cytosines shown in *green*, and a conserved water molecule shown in *purple. C,* representative EMSA analysis of wild type (*left panel*), S334E phosphorylation mimic (*right*), and PKA-phosphorylated Etv1 (*center panel*) titrated against normal DNA substrate. The DNA binding isotherms derived from these data are plotted below. *D,* model of potential clashes between phosphorylated Etv1 Ser-334 in the Etv1-dsDNA structure (PDB code 4BNC) superimposed onto a longer dsDNA from PU.1-dsDNA (PDB code 1PUE). Distances between potentially clashing atoms are shown as *green dashed lines*.

To test whether CpG methylation directly affects Etv1 binding, we modified the 20-bp oligonucleotides used in the EMSA experiments by substituting 5meC at position −1 of the consensus (CGGAA) along with the cytosine complementary to G+1, generating a fully methylated CpG motif (C^m^GGAA/TTCC^m^G). EMSA experiments showed that the Etv1 protein failed to bind the methylated DNA (Fig. 7*A*). Modeling of 5meC at these positions in the Etv1-DNA and Fev-DNA structures (Fig. 7*B*) showed Arg-394/106 (Etv1/Fev) was close to the 5meC at −1 of the CGGAA consensus strand, and Asp-387/99 was close to 5meC at both −1 of the CGGAA strand and +1 on the complementary strand. Methylation of cytosines at these positions could create a steric clash with Arg-394/106 causing loss of hydrogen bonds involved in the conserved water network. EMSA analyses of Etv1^D387A^ and Etv1^R394A^ mutants showed that replacement of either of these charged side chains with the smaller and hydrophobic alanine recovered some binding to methylated DNA (3 and 2% of total DNA bound, respectively, at the highest protein concentration) compared with the wild-type protein (Fig. 7*A*). It may be significant that Asp-387 (or glutamate) is almost invariant in the ETS recognition helix, with only class III proteins displaying a substitution to glutamine (32). As class I Ets proteins predominantly select cytosine at the −1 position of the consensus, although class III members typically have adenine (32), it is possible that Asp-387 (or Glu) plays a universal role not only in the preference for cytosine at −1 but also to the discrimination against CpG-methylated sites.

##### Direct Regulation of DNA Binding by PKA Phosphorylation of the Etv1 Ets Domain

Ets proteins are regulated by phosphorylation (68); for example, the protein kinases RSK1 and PKA phosphorylate Etv1 at multiple positions *in vivo* (40). PKA phosphorylation of Etv1 Ser-334 (at the N-terminal edge of the Ets domain) inhibits *in vitro* DNA binding of the full-length protein but increases transcriptional transactivation potential, as does phosphorylation of the equivalent Ser-367 in Etv5 (41). In contrast, Etv4 lacks a serine at the equivalent position (*asterisk,* Fig. 1*A*). To assess whether Ser-334 phosphorylation of the Ets domain affects DNA binding directly, we phosphorylated Etv1 with PKA on Ser-334 *in vitro* (Fig. 7, *C* and *D*). In parallel, we produced a phosphomimetic mutant of Ser-334 to glutamate. EMSA experiments showed that phosphorylation of Ser-334 reduced the binding affinity by at least 200-fold (Fig. 7*C*). This confirms that Ser-334 phosphorylation can directly interfere with DNA binding of the Etv1 Ets domain, independent of other regions of Etv1 or of other proteins (40). Interestingly, the S334E mutation caused only a modest reduction in the binding constant, but overall probe binding levels were 35% at apparently saturating DNA concentration. This may indicate a kinetic instability of DNA binding by the mutant protein. A phosphoserine residue is bulkier and carries more negative charge than glutamate, suggesting that the strong effect of the phosphate is due to charge repulsion. Modeling of a phosphorylated Ser-334 in the Etv1-dsDNA structure (PDB code 4BNC) superimposed onto a longer dsDNA (Fig. 7*D*) shows a possible repulsive interaction between the phosphate on the protein and the DNA backbone, which may underlie the decrease in DNA binding.

##### Etv1/4/5 and Fev Are Disulfide-linked Homodimers

**FIGURE 8.**
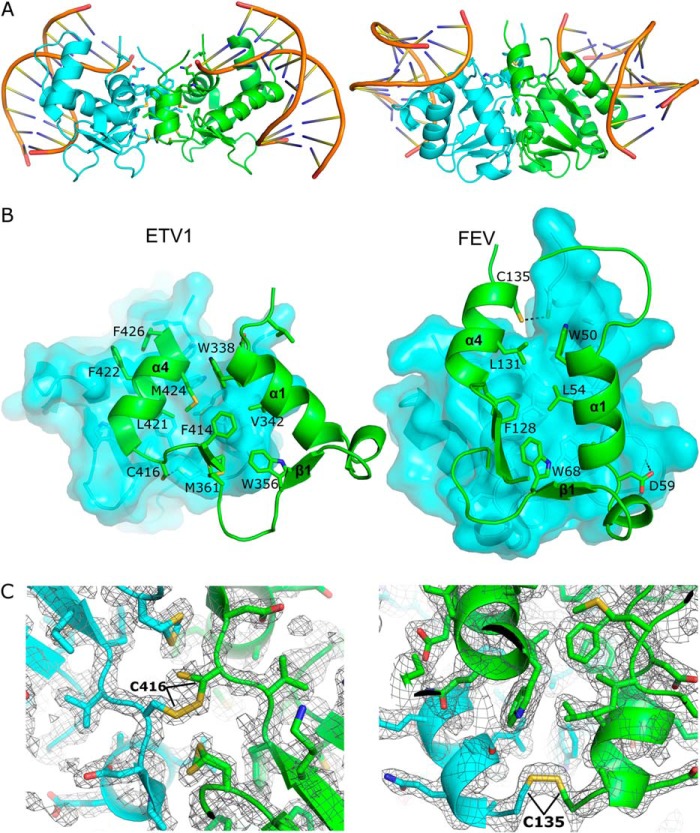
**Overview of the dimerization interfaces.** *A,* view of the potential dimers formed in Etv1 (shown on the *left*) and Fev (*right*) DNA complexes with the 2-fold axis vertical, showing the DNA in cartoon representation. The structures of the Etv4 and Etv5 dimers are essentially identical to Etv1. *B,* close view of the secondary structural elements and residues that compose the dimer interfaces for Etv1 (*left*) and Fev (*right*) with polar contacts depicted as *dashed lines. C,* 2*F_o_* − 1*F_c_* electron density maps, for Etv1 (*left*) and Fev (*right*), both contoured at 1.0 σ in the vicinity of the intermolecular disulfide bond. Two conformations can be seen for Cys-416 in the Etv1 structure (also observed in the high resolution structures of Etv4 and Etv5), although it is not clear whether this is induced by radiation damage or an intrinsic property of this residue.

Analysis of the contacts between adjacent molecules in the Etv1, Etv4, and Etv5 crystals reveals a conserved potential dimer interface that is distant from the DNA binding surface and is present in all the DNA-free and DNA-bound crystal forms (Fig. 8*A*). This interface is centered around contributions from the N- and C-terminal helices, α1 and α4, with additional contacts involving residues from the β1-β2 loop (Fig. 8*B*). The contact area for this interface is ∼800 Å^2^, includes contributions from 20 residues, and accounts for the burying of 15% of the monomer accessible surface area upon complex formation. The majority of the interface is nonpolar in nature with extensive hydrophobic interactions. Similarly, an analysis of the crystal contacts in both the Fev and Fev-DNA crystals reveals an interface that includes the same components (α1 and α4 and the β1-β2 loop) as that of the other three Etv proteins. However, the dimer interfaces in Etv1/4/5 and in Fev are not equivalent; rather, they differ by a relative rotation of ∼90°, reflecting the different orientations of the α4 helix (Fig. 1*C*). The contact area for the Fev dimerization interface is ∼700 Å^2^, spans 21 residues, and accounts for 12.5% of the monomer accessible surface area. Similar to Etv1, the interface is dominated by contacts from hydrophobic residues, a single hydrogen bond (Asp-59–His-72), and an intermolecular disulfide bond between Cys-135 and its symmetry mate (Fig. 8, *B* and *C*). All the proteins were purified and crystallized in the presence of reducing agent (1 mm TCEP), although the reducing agent may have become oxidized during crystallization in an oxygen-containing environment.

**FIGURE 9.**
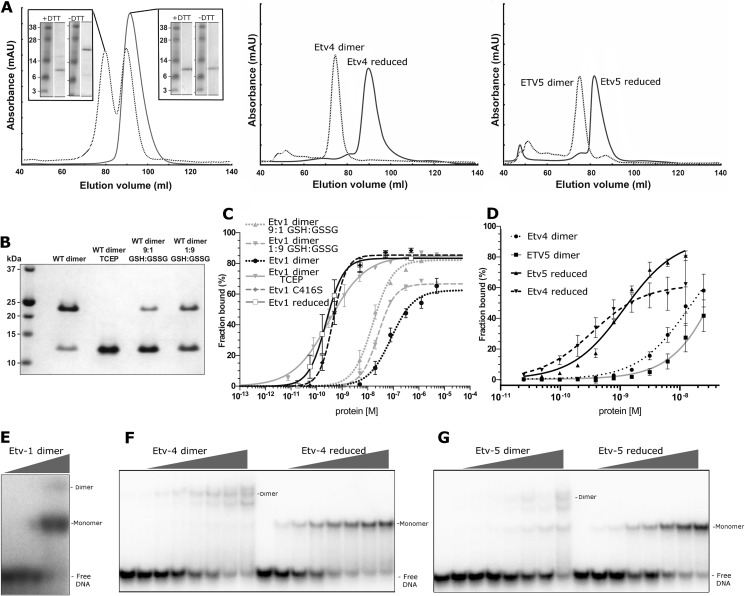
**Redox-dependent regulation of Etv1, Etv4, and Etv5.** *A,* purification of Etv1 (*left panel*), Etv4 (*center panel*), and Etv5 (*right panel*) under reducing (*solid lines*) or nonreducing conditions (*dashed lines*). The *inset in the left-hand panel* shows the SDS-PAGE analysis of the size exclusion fractions denatured in the presence (*upper right*) or absence (*lower right*) of 5 mm DTT. *B,* nonreducing SDS-PAGE of an SEC fraction of Etv1 eluting as a dimer (WT dimer) or treated prior to gel loading under various reducing conditions: 10 mm TCEP, and different ratios of reduced (GSH) and oxidized (GSSG) glutathione. *C,* DNA binding isotherms of different oligomeric/redox states of Etv1 from EMSA analysis. The proteins used were as follows: Etv1 purified under reducing conditions (monomeric); Etv1-C416S mutant (monomeric); Etv1 dimer purified under oxidizing conditions; Etv1 dimer treated with 10 mm TCEP; Etv1 dimer treated with reduced glutathione: and Etv1 dimer treated with oxidized glutathione. *D,* DNA binding isotherms of Etv4 and Etv5 from EMSA analysis. The proteins used were as follows: Etv4 purified under oxidized conditions (dimeric); Etv5 purified under oxidized conditions (dimeric); Etv4 dimer treated with 10 mm TCEP; and Etv5 dimer treated with 10 mm TCEP. DNA probe concentration was 0.2 nm. *Error bars* are plotted as ± S.E. *E,* EMSA of dimeric Etv1 showing a slower mobility Etv1-DNA species that is presumed to represent the dimeric species. *F,* EMSA of dimeric and reduced Etv5 showing bands representing monomeric and dimeric DNA complexes. *G,* EMSA of dimeric and reduced Etv5 showing bands representing monomeric and dimeric DNA complexes.

Intermolecular dimerization has been documented previously in Ets factors (69), with ETS1 forming head-to-head homodimers required for cooperative binding on palindromic and other sites (70). ELK1 dimerization appears to regulate ELK1 cellular stability (71), with a homotypic interface utilizing the α1 helix, similar to that of Etv1/Fev, but with a different positioning and orientation (65). The presence of an intermolecular disulfide bond formed between similar C-terminal regions Etv1, Etv4, Etv5, and Fev is particularly significant, as they are homologous to ETS1 that can form disulfide-linked dimers *in vivo* (72). Although present in the crystal structures, disulfide-linked Ets domain dimers were not initially observed in solution (Fig. 9*A*). When Etv1, Etv4, or Etv5 was expressed in Rosetta-gami^TM^ 2 *Escherichia coli* (73) and purified under nonreducing conditions, a peak consistent with the dimer molecular weight was observed in size exclusion chromatography (Fig. 9*A*). SDS-PAGE in the presence or absence of DTT suggests the higher molecular weight peak contained a reducing agent-sensitive dimer, which was further confirmed by mass spectrometry. We have been unable to test whether Fev also forms disulfide-linked homodimers, as our Fev constructs did not produce soluble protein in the Rosetta-gami^TM^ 2 *E. coli* cells.

##### Disulfide Bond Formation and Dimerization Inhibit DNA Binding

To discover the functional consequences of disulfide-dependent dimerization, we compared the DNA binding affinities of purified monomeric and dimeric Etv1 using EMSA. The reduced monomeric Etv1 protein bound the DNA probe with an affinity up to 200-fold higher than the oxidized dimeric form (Table 3; Fig. 9, *A* and *C*); a C416S mutant of Etv1 bound DNA with an affinity similar to the reduced wild-type Etv1 (0.26 nm). Similarly, dimeric Etv4 and Etv5 also bind DNA with affinities that are 2 orders of magnitude lower than the reduced monomeric forms (Fig. 9, *D*, *F,* and *G* and Table 3; note that the *K_d_* values of the dimeric forms are rough estimates, as saturation could not be reached). A graded response to physiological redox potentials could be seen when Etv1 dimers were treated with different ratios of reduced and oxidized glutathione; the extent of DNA binding roughly correlates with increasing the reducing agent potential and dimer dissociation (Fig. 9, *B* and *C*). We conclude that Etv1, Etv4, and Etv5 binding to DNA is reversibly inhibited by the formation of a disulfide-linked dimer.

DNA complexes of dimeric Etv4 and Etv5 migrate at a lower mobility than complexes of the monomeric proteins (Fig. 9, *F* and *G*), indicating the presence of a dimer of Etv4/5 with one or two bound probe molecules. EMSA using the dimeric form of Etv1 results in a band with mobility of a monomer (Fig. 9*E*); we assume this reflects the small amount of monomeric protein that is present in the high molecular weight SEC fractions (Fig. 9*B*, *left lane*); this monomeric subpopulation could not be removed by repeated chromatography. However, at the highest concentrations of dimeric Etv1 and DNA, a weak slower migrating band could be observed with a mobility similar to that of the Etv4 and Etv5 dimeric complexes. The paradoxical observation of the dimer in complex with DNA in the various crystal structures may be accounted for by high concentrations of both protein and DNA (∼1 mm) during crystallization, which may be well in excess of the dissociation constant. We also note that both DNA-bound and -unbound forms of the proteins crystallized in the disulfide-bonded form, perhaps indicating that the monomeric form is considerably more flexible and difficult to crystallize.

## Discussion

Although the interaction between Ets domains and DNA has been the subject of numerous previous structural and biochemical studies (32, 74), the question of how Ets domains are able to achieve DNA sequence specificity beyond the GGA consensus motif has remained open. Furthermore, although a variety of post-translational modifications regulate Ets transcription factors (39, 40, 75–77), the mechanisms involved have yet to be unambiguously clarified (8). In particular, it is not known whether modification exerts a direct effect on Ets protein-DNA binding, protein-protein interactions, or stability.

Our structural analysis of the Etv1, Etv4, Etv5, and Fev Ets domains both in the presence and absence of DNA has allowed us to identify additional features of the protein-DNA interface. Mutation of individual residues supports predictions from the crystal structure for binding the GGA core for most residues tested. Remarkably, mutating any of the residues of Etv1, which interact directly with the DNA backbone (Lys-379, Tyr-396, Tyr-397, and Lys-404), led to complete loss of binding. The extent to which these substitutions affect the binding affinity indicates that these residues might play a crucial role in opening the DNA to allow the α3 recognition helix to access the widened major groove or anchor the Ets domain once recognition has occurred. Further interface features include the dynamic nature of a conserved tyrosine (Tyr-395 in Etv1), which may allow for recognition for up to three bases downstream from the GGA core. In addition, a conserved cluster of coordinated water molecules supports a structural basis for sequence recognition of two bases upstream of the GGA core, including selectivity against thymine as well as 5-methylcytosine bases at position −1 of the consensus motif. Methylated bases may create an energetically unfavorable environment for conserved aspartate and arginine residues, preventing Ets proteins from binding when transcription is to be repressed by CpG methylation (43), as observed previously for Ets proteins (44–46). Although difficult to rule out the role of indirect recognition through sequence dependence of the DNA bending observed in our structures, the additional direct and water-mediated interactions seen in the Etv1 and Fev protein-DNA crystal structures appear to be sufficient to explain almost entirely the observed sequence specificities.

Etv1 is post-translationally regulated by Rsk1 and PKA phosphorylation *in vivo* (40). We were able to reproduce the inhibition of DNA binding by specific phosphorylation of Ser-334, a known PKA target site, in the isolated Ets domain *in vitro*. This demonstrates a direct effect of post-translational regulation on an Ets domain, and our Etv1-DNA structure suggests that an increase in negative charge around Ser-334 following phosphorylation may directly abrogate DNA binding following electrostatic clashes.

Crystal structures of all four Ets domains exhibit disulfide-linked homodimers. Dimerization of Etv1, -4, and -5 is mediated by the homologous cysteine residues (Cys-416, Cys-422, and Cys-449, respectively). Fev dimers are linked by a different, nonconserved cysteine (Cys-135).

**FIGURE 10.**
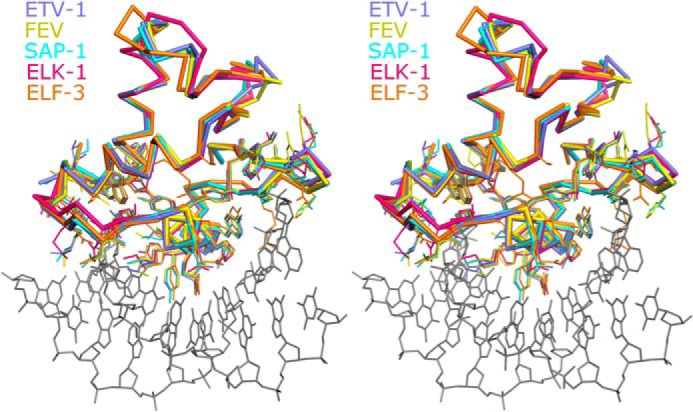
**Comparison of the overall features of DNA binding of Etv1 and Fev with other high affinity Ets DNA complexes.** Stereo view of a structural superposition of Etv1 (*blue*) and Fev (*yellow*) (which are in the inhibited form) with three other noninhibited Ets DNA complexes SAP-1 (*cyan*), ELK-1 (*pink*), and ELF-3 (*orange*). A single DNA molecule (taken from the Etv1-DNA complex) is shown in the *stick format* for reference.

We found that disulfide-mediated dimerization strongly inhibited Etv1, Etv4, and Etv5 DNA binding *in vitro*, which was directly reversible with increasing redox potential. Our crystal structures do not provide an explanation for this inhibition; the DNA in the crystal is bound to the dimeric protein, and the dimerization interface is sufficiently distant from the DNA binding interface to eliminate the possibility of steric effects with the substrates used (20-mer with the consensus sequence centrally located). Examination of the protein DNA interfaces in these crystals and comparison with other active Etv DNA complexes reveal very similar interfaces, with similar contacts made to the phosphodiester backbone and the major sequence-specific DNA interactions provided by the recognition helix being totally conserved (Fig. 10). Thus, we believe the interfaces in our dimeric crystal structures to be representative of the higher affinity monomer DNA interface. This leads us to suggest that that the most likely explanation for the inhibition caused by dimerization is an allosteric mechanism of inhibition, which acts through changes in protein flexibility or dynamics. The mechanism may be similar to the autoinhibitory mechanism of ETS1, where packing of a 4-helical inhibitory module also distal to the DNA-binding face allosterically modulates subtle structural changes that inhibit DNA binding (78). It is also possible that the constraints of crystallization favor a protein conformation that is less stable in solution. However, structural studies of allosteric regulation do not always reveal a clear-cut mechanism, and there are cases where allosteric regulation acts through changes in protein flexibility or dynamics (79–81).

Redox control of transcription factors is a recognized regulatory mechanism (80, 82–84). Examples include intermolecular disulfide bridges in bZIP proteins, such as AP-1 (85), and cysteines outside the DNA-binding motif mediating redox-sensitive dimerization in plant homeodomains (86). Ets proteins have been implicated in redox signaling *in vivo*, such as the oxidative inactivation of GABPα by the Hippo pathway (87). The mechanism of redox-dependent DNA binding inhibition in Ets factors has remained unclear, as many contain multiple cysteine residues. Here, we show that a single redox-sensitive cysteine is sufficient to confer inhibition of DNA binding by dimerization.

Notably, cysteines at positions equivalent to Etv1-Cys-416 exist in addition only in GABPα, ETS1, and ETS2 out of all human Ets domain proteins. Although dimerization and disulfide formation seem to have regulatory influence on proteins, including GABPα (88) and ETS1, the equivalent cysteines in crystal structures of ETS1, GABPα, and ETS2 (89, 90) do not seem to be involved in intermolecular disulfide bridges. This indicates that the precise mode of redox regulation seen for Etv1/4/5 may be restricted to a small subset of the Ets protein family.

Etv1, -4, and -5 are strongly implicated in cancer (8). Our data suggest the possibility that redox-mediated dimerization could link Etv1/4/5 factors to the response of cancer cells to their microenvironment. If proven, chemoprevention by targeting such redox-sensitive transcription factors could potentially deliver novel therapeutic strategies (91).

### 

#### 

##### Protein Data Bank Accession Numbers

Atomic coordinates and structure factors were deposited in the Protein Data Bank with accession numbers 4AVP (Etv1), 4BNC (Etv1-DNA), 4CO8 (Etv4), 4UUV (Etv4-DNA), 4UNO (Etv5-DNA), 2YPR (Fev), and 3ZP5 (Fev-DNA).

## Footnotes

5 The abbreviations used are:

EBS: Ets-binding site
5meC: 5-methylcytosine
TCEP: tris(2-carboxyethyl)phosphine
BisTris: 2-[bis(2-hydroxyethyl)amino]-2-(hydroxymethyl)propane-1,3-diol
MWCO: molecular weight cutoff
CV: column volume
PDB: Protein Data Bank
SEC: size exclusion chromatography
TEV: tobacco etch virus
GABP: GA-binding protein α.
